# Tissue response to applied loading using different designs of penile compression clamps

**DOI:** 10.2147/MDER.S188888

**Published:** 2019-06-27

**Authors:** Joseph MH Lemmens, Jackie Broadbridge, Margaret Macaulay, Rowland W Rees, Matt Archer, Marcus J Drake, Katherine N Moore, Dan L Bader, Mandy Fader

**Affiliations:** 1 University of Southampton, School of Health Sciences, Southampton SO17 1BJ, UK; 2 Department of Urology, University Hospital Southampton NHS Foundation Trust, Southampton, SO16 6YD, UK; 3 Bristol Urological Institute, Southmead Hospital, Bristol, BS10 5NB, UK; 4 Faculty of Nursing, University of Alberta, Edmonton, Alberta, T6G 1C9, Canada

**Keywords:** penile, compression, clamp, urinary, incontinence, pressure

## Abstract

**Background:** Penile compression devices (PCD) or clamps are applied to compress the urethra and prevent urinary incontinence (UI). PCDs are more secure and less likely to leak than pads, allowing men the opportunity to participate in short-term, vigorous activities. However, they are uncomfortable, can cause pressure ulcers (PU) and affect penile blood flow. No objective assessment of tissue health has been undertaken to assess and compare different PCD designs and to provide guidance on safe use.

**Objective:** This study was designed to evaluate existing PCDs in terms of their physiological response and potential for pressure-induced injury.

**Design, setting and participants:** Six men with post-prostatectomy UI tested four selected PCDs at effective pressures, in a random order, in a controlled laboratory setting.

**Outcome measurements and statistical analysis:** Using objective methods for assessing skin injury, PCDs were measured in situ for their effects on circulatory impedance, interface pressures and inflammatory response.

**Results and limitations:** There was evidence for PCD-induced circulatory impedance in most test conditions. Interface pressures varied considerably between both PCDs and participants, with a mean value of 137.4±69.7 mmHg. In some cases, penile skin was noted to be sensitive to loading with elevated concentration of the cytokine IL-1α after 10 mins wear, indicating an inflammatory response. IL-1α levels were restored to baseline 40 mins following PCD removal.

**Conclusion:** Skin health measures indicated tissue and blood flow compromise during the 50 mins of testing using all PCDs. Although there was an elevation in pro-inflammatory cytokines, PCDs did not cause sustained irritation and skin health measures recovered 40 mins after PCD removal. This research indicates that application of a clamp for one hour with an equal clamp free time before reapplication is likely to be safe. Longer periods are often recommended by manufacturers but have yet to be tested.

## Introduction

Urinary incontinence (UI) is a complication suffered by men who undergo surgery for prostate cancer. This debilitating condition has a major effect on quality of life, with an impact considered greater than erectile dysfunction (ED),^1^ affecting personal and professional relationships, and can lead to depression and social isolation.^2^–^5^ Although the majority of men recover continence within 12 months, about 15% will be affected throughout their lifetime and require non-surgical methods to manage the leakage.^6^ Incontinence pads represent a common management choice but they have many drawbacks. They can be bulky, hot, difficult to dispose of, require considerable storage space and many men consider them to have an unacceptable “babyish or feminine” appearance.^7^ Sheaths, body-worn urinals (BWU) and PCDs are alternatives or adjuncts to pads. Of these, PCDs have been reported to be particularly useful for certain activities when a secure, discreet device is required.^8^ However, little has been published on the correct use or applied pressures necessary for these devices^8^,^9^ and if used improperly, PCDs can place the individual at risk for penile trauma. Such a lack of evidence on safety is a barrier to their use. Lack of prescribing means that appropriate candidates are prevented from using a potentially effective short-term continence option. Moreover, it may mean that men purchase PCDs online and use them without professional support and advice, with the potential risk of penile injury in the form of medical-device related pressure ulcers (MDRPU).

Only two studies have evaluated PCDs, in terms of penile blood flow and user acceptability.^8^,^9^ The first reported that the Cunningham PCD was the most effective of three PCDs at significantly reducing leakage, with an associated decrease in penile blood flow from 125 to 73 mm/s.^9^ In the other larger study, the Cunningham PCD was also reported to be the most effective device at reducing leakage and being secure, but was more painful compared with non-compression products ie, sheaths, BWUs and pads. Those who could tolerate the PCD found it to be useful for short vigorous activities such as swimming or dancing.^8^ Although neither study reported on pressure-related injury, several case reports^10^,^11^ attest to the risk of mechanically induced penile soft tissue injury and the formation of pressure ulcers.

Currently, there is insufficient research on PCDs to allow evidence-based decisions by clinicians and consumers. It appears that current products are limited by discomfort and urine leakage as well as a potential for penile injury and reduced penile blood flow. There is a role for a safe, effective clamp to augment continence management, but increased understanding is required of the effects on penile perfusion and skin health for existing PCDs before users and HCPs can prescribe them with confidence.

This study represents part of a larger research programme to investigate the performance of PCDs. It utilizes established objective bioengineering techniques to assess the effects of applied loading on skin response incorporating four commercially available PCDs, which are applied at a pressure sufficient to restrict incontinence.

## Aims and objectives

### Aim

To evaluate the tissue response to different commercially available PCDs with a view to assessing potential risk of their use.

### Objectives

To evaluate applied interface pressure, circulatory impedance and inflammatory response when PCDs are applied at relevant pressures necessary to reduce or eliminate UI in men post-prostatectomy.

## Materials and methods

### Participants

Experienced PCD users with post-prostatectomy UI were recruited from previous participants,^8^ prostate cancer support groups and incontinence organizations. The inclusion criteria included men with UI for greater than 12 months after radical prostatectomy, who had experience of using a PCD. The exclusion criteria included the self-reported absence of sensation to the penis, urgency or urgency incontinence, ie, a strong uncontrollable urge to pass urine and leakage before reaching the toilet, as the predominant urinary symptom. In addition, men were excluded with a poor memory or forgetfulness (Mini Mental State Exam <27) leading to an inability to reliably release the PCD regularly at least every 2 hrs, and those in the terminal stages of an illness.

Ethical approval was given by the South Central Hampshire B Research Ethics Committee (14/SC/1034, 05.09.14) and informed written consent was obtained from each volunteer. The study was conducted in accordance with the Declaration of Helsinki.

### Devices

**Figure 1:**
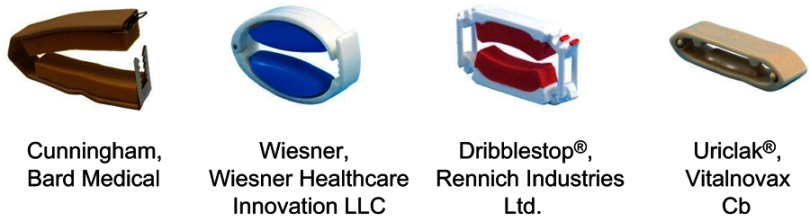
PCDs selected for laboratory testing.

Samples of PCDs available worldwide (n=14) were obtained. A group of experienced PCD users tried each PCD for 30 mins, while also wearing a pre-weighed pad, during a standard exercise regime. Leakage was assessed based on subtraction of the pre-test pad weight from the post-test pad weight. A subset of four PCDs was selected for laboratory testing representing the main designs, while being both the most effective at preventing urine loss in the pad test and the most acceptable to users (Figure 1).

### Participant testing and procedures

As blinding was not possible, PCDs were tested in random order determined through use of Latin squares.^12^ A series of tests was performed for each PCD with each participant to measure applied interface pressure, circulatory impedance and inflammatory response. After baseline measurements were recorded in the absence of a device in situ (Time 0), participants applied the PCD to a tightness which they judged from previous experience to be tolerable and effective, termed the prescribed interface pressure (PIP). Measurements were then recorded during wear 10 mins and 50 mins after application, immediately after removal, ie, 55 mins after application, and after a 40 mins recovery period, ie, 90 mins after application (Table 1).

**Table 1:** Laboratory testing timeline for measurements

Timeline (minutes) →	Baseline (no PCD)	PCD applied (“wear time” =50 mins)			PCD removed (“rest time” =40 mins)	
0	10	50	55	90
Test →	CI; IR	IP	CI; IR; IP	CI; IR; IP	CI	CI; IR

**Abbreviations:** PCD, penile compression device; CI, circulatory impedance; IR, inflammatory response; IP, interface pressure.

## Test outcomes

### Applied interface pressure

**Figure 2:**
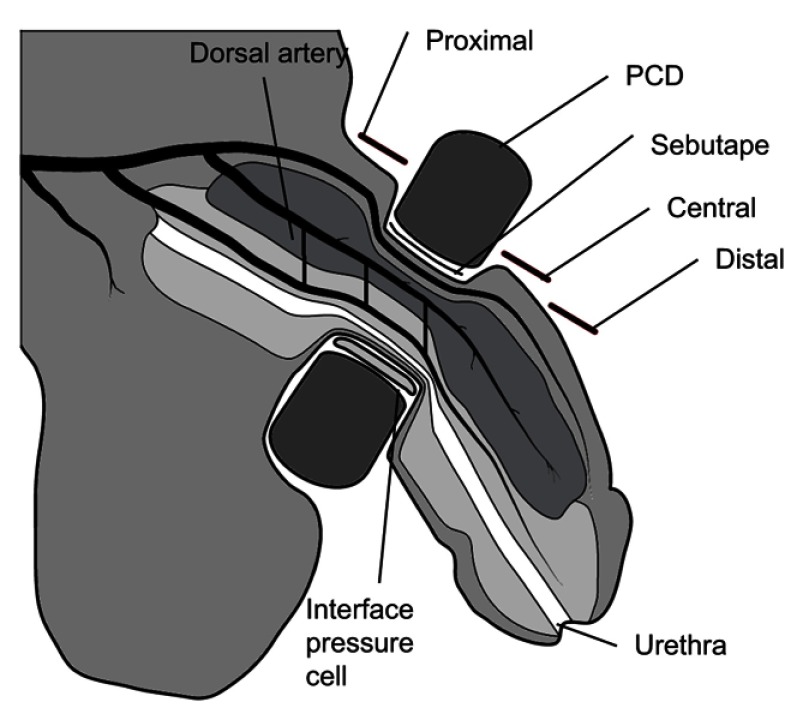
Location of the three measurements to assess the skin response on the penis when subjected to each of the penile compression devices (PCD) applied at their associated prescribed interface pressures.

Applied interface pressures between the PCD and the soft tissues of the penis were measured, using an established commercial system (Talley Mk3 Pressure Monitor, Talley Medical, UK). This electro-pneumatic based system has a reported mean error of 12±1%.^13^ The measurement involved positioning an individual air cell, 18 mm in diameter, on the ventral surface of the penis under the arms of the PCD (Figure 2).

### Circulatory impedance

Laser Doppler perfusion imaging (LDPI) represents an established method for assessment of penile perfusion^9^ and circulatory impedance. A commercial system (LDI2-VR, Moor Instruments, UK), activated at a wavelength of 633 nm at a power of 2.5 mW, was used to measure blood flux in arbitrary units (AU) at the dorsal surface of the penis within three regions of interest (ROI), namely, proximal, central and distal (Figure 2) to the PCD at a sub-dermal depth of approximately 0.6 mm.^14^

### Inflammatory response

Sebutape^®^ (CuDerm Corporation, Dallas, Texas, USA) represents a hydrophobic lipid absorbent micro-porous film used to collect skin sebum. It has been previously employed to examine a number of pro-inflammatory cytokines, using a well-established protocol.^15^,^16^ A Sebutape sample, 400 mm^2^ in area, was placed on the dorsal side of the penis between the PCD and the skin and the PCD was applied at the PIP for 2 mins (Figure 2). Samples were then carefully removed using blunt forceps and placed in plastic low-bind 2 mL microtubes (Axygen™ MaxyClear), which were then placed on ice and frozen at −80°C. Collections using fresh Sebutape were repeated throughout PCD wear and recovery periods (Table 1).

### Biochemical analysis

Frozen Sebutapes^®^ were rapidly thawed to room temperature and 1.7 mL of phosphate buffered saline (PBS; Sigma-Aldrich Co., St Louis, US) solution was added to each vial. After 1 hr, the tapes were sonicated for 10 mins at 20°C, vortexed vigorously for 2 mins and additionally mixed with a pipette tip. After refreezing overnight at −80°C, the tape extracts were thawed, vortexed for 1 min and mixed with a pipette to recover the total extracts from the tapes. Extracts were aliquoted into five vials (Thermo Scientific™ Low Retention). Samples from all participants were then processed and analyzed in triplicate using immunoassay kits (PeproTech ELISA ABTS kits, New Jersey, USA) to estimate concentrations of IL-1α with a detection range of 8-1,000 pg/mL. Sample dilutions ensured the detection was extended up to 4,000 pg/mL.

### Data analysis

Descriptive and inferential statistics were performed using Microsoft Excel. Non-parametric descriptors were used for cytokine concentrations. Due to the small size, a Wilcoxon Rank Sum Test was used for paired cytokine measures and to evaluate the effects of clamping pressure on blood perfusion flux. Statistical significance was defined as *p*<0.05.

## Results

### Participants

Six experienced clamp users were recruited into the study, with a mean age of 71.5±4.97 years and a mean BMI of 26.45±3.14 kg/m^2^.

### Interface pressure

**Figure 3:**
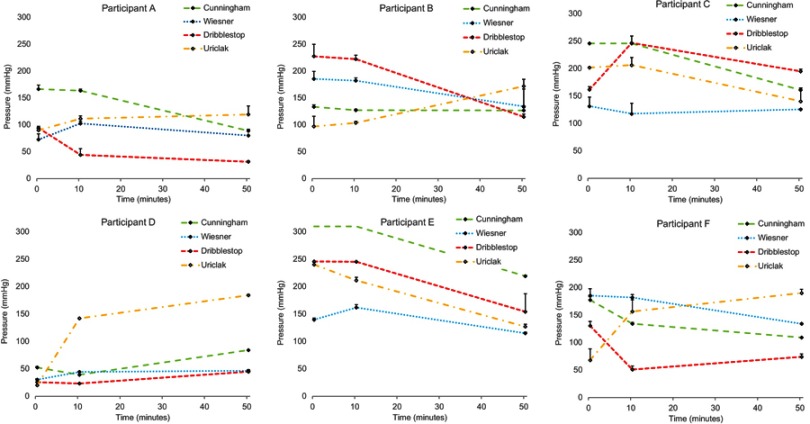
Temporal profiles of mean interface pressure values ± SD for six participants and each of the penile compression devices ( PCDs) over the 50 mins wear period.

PIP varied considerably with a mean value of 137.4±69.7 mmHg, thus precluding any inter-participant comparisons. Figure 3 shows the mean interface pressures measured at 0, 10 and 50 mins during the wear period for each PCD and participant. In many cases, there was a moderate variation in interface pressure values over the 50 mins wear period. It is noteworthy that there was a decrease between 10 and 50 mins wear for the Dribblestop PCD (Figure 3B and 3E), compared to a corresponding temporal increase for the Uriclak PCD (Figure 3D and 3F).

### Circulatory impedance

**Figure 4:**
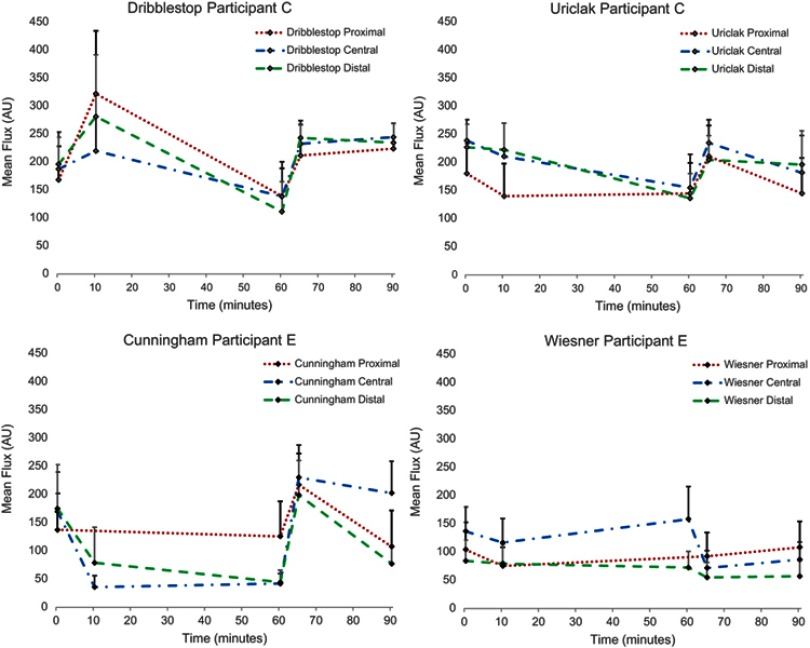
Temporal profiles of Doppler ROI values in three regions of the penis of two participants for each of the penile compression device (PCD) designs. Error bars represent perfusion flux SD.

**Figure 5:**
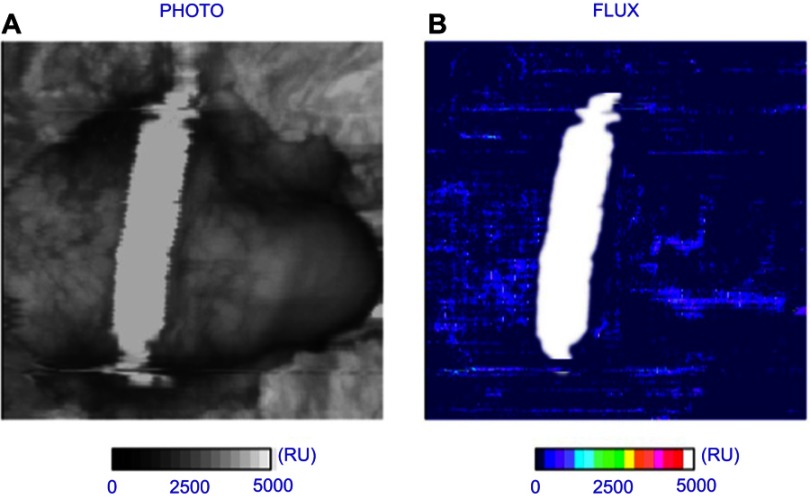
The Dribblestop penile compression device (PCD) had, by 60 mins wear time, decreased distal blood perfusion flux (Figure 4) after an initial rise corresponding to a sustained increase in interfacial pressure (Figure 3). (**A**) Scanned image (**B**) PCD location superimposed over flux scan.

There was a small change in perfusion for each PCD over the 50 mins wear period. This is illustrated with two of the participants in Figure 4. The Cunningham PCD caused the most significant decrease in blood perfusion, with values below 50 AUs in both central and distal ROIs. Figure 5 illustrates the decrease with the Dribblestop PCD. The effect of removing the PCDs was estimated by examining the perfusion values during the first 5 mins of the recovery period. It is evident that removal of three of the PCDs (Cunningham, Uriclak and Dribblestop) resulted in an increased perfusion, indicating a reactive hyperemic response following circularity occlusion. By contrast, the Wiesner PCD exhibited only small changes during both the wear and recovery periods.

There were noticeable differences in perfusion values between the different ROIs. For example, by assuming unequal variances, a statistically significant difference was found between pressure and mean perfusion flux within the central ROI (*p*≤0.01). In addition, there was a statistically significant difference between values at baseline and after 10 mins of wear in the distal ROI (*p*≤0.01) alone.

Following release of the PCD at the end of the wear period, there was a significant difference in perfusion at the proximal ROI only (*p*<0.05). No significant differences were evident between the end of the recovery period and baseline values for all three ROIs, suggesting that flow returns to normal after a 40 mins recovery period.

### Inflammatory response

**Figure 6:**
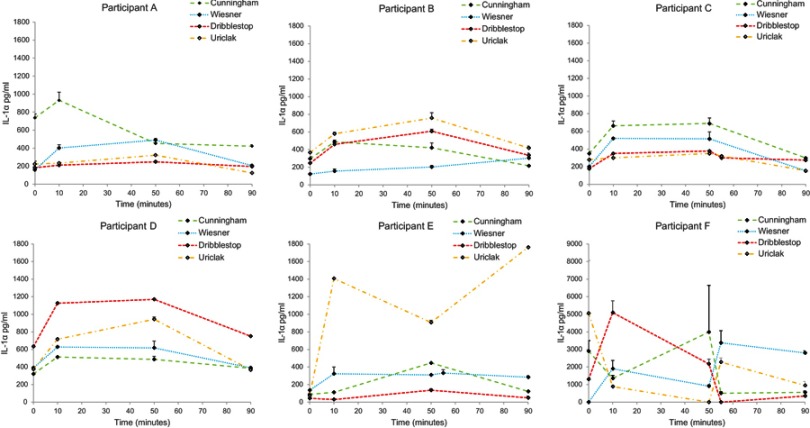
Inflammatory response (IL-1α [pg/mL]) measured at the skin surface over the 90 mins test period.

Figure 6 reveals considerable intra-individual variability in IL-1α concentrations. Nonetheless, four of the participants (A, B, C and D) produced similar IL-1α concentration profiles with a general increase over baseline at 10 mins, which was maintained throughout the wear period. Following the 40 mins recovery period, cytokine levels had returned to baseline, resulting in no significant differences between their values and those of baseline (*p*>α=0.05).

Although participant E, who presented with a significant penile retraction, reported a preference for the Uriclak PCD, this design yielded a higher release of IL-1α than the other PCDs. The Cunningham PCD was applied at the highest pressures in excess of 250 mmHg (Figure 3E). Immediately after removal, an IL-1α concentration value beyond the limit of the IL-1 detection was obtained.

## Discussion

This study was designed to evaluate four existing PCDs for penile physiological response and potential for pressure-induced injury. With pressures adequate to prevent UI in male volunteers, we found reduced circulatory impedance and raised superficial inflammatory response, as reflected in an up-regulation of IL-1α. These raised levels indicate a potential for skin and soft tissue injury depending on wear time and applied pressure and suggest that design improvements are warranted. We did not find that overall any one design produced a less adverse impact on physiological measurements than the others.

A computer model confirms the limitations of current PCD designs and the potential risk of soft tissue damage.^17^ Interface pressure of 188 mmHg within the range of the present in vivo study (Figure 3) caused internal stresses greater than 75 mmHg in the skin, fat and tunica albuginea proximal to the urethra. Adjusting the model to reflect slight changes in the angle of the PCD, as would occur in normal wear, resulted in an increase in tissue strains/deformations and tissue stresses. The highest stress on the urethra was predicted with the Wiesner PCD, a design which utilizes a rigid knurl behind a silicone cover (Figure 1). In our study, the Wiesner did not generate significantly different pressures or circulatory impedance when compared to the other designs but when it was trialed at home, men reported more discomfort and one incident of haematuria.^18^

Each PCD restricted venous return to some extent resulting in blood pooling distally and engorgement of the penis. However, three of the PCDs (Dribblestop, Cunningham and Wiesner) loosened during wear and required adjustment as evidenced by the small reduction in interface pressures. This may be a result of various factors including the viscoelastic behavior of soft tissues,^19^ softening of the erectile state and possible reduction in the flexural modulus of the device materials due to body heat and moisture. By contrast, the Uriclak PCD, which operates using a sprung metal section (Figure 3D), did not loosen with wear and its continued use resulted in increased interface pressures in four of the six participants.

Compression of blood vessels by the PCDs affected penile circulation as measured by Doppler perfusion flux (Figure 5). Importantly, on removal, perfusion levels were restored to unloaded levels after the 40 mins recovery period. Although three of the participants (Figure 3: B, C, E) recorded pressures in excess of 250 mmHg, the corresponding mean perfusion values were also high (Figure 4). This anomaly might be explained by compression causing partial constriction of a number of blood vessels within the network with a corresponding increased flow within the remaining vessels. However, this finding could still indicate total under-perfusion in the vulnerable penile tissues, as the network of capillaries is copious and there may be hypoxia within the deep tissues of the corpora cavernosa.

Potential pressure injury to the penis from impaired circulation has been studied in cyclists. Compression by bike saddles has been associated with perineal and penile trauma including neural injury, erectile dysfunction and numbness. In one small cohort study, MR images revealed large mean compressive strains of 56% and 67% in the corpus spongiosum and the corpus cavernosum, respectively.^20^ These strains were associated with mean anterior and posterior saddle pressures of 110 mmHg and 146 mmHg, respectively, values similar in magnitude to those measured in the present study.

As pressures from the PCD may cause ischemia, there could be cellular damage from a reduction in tissue oxygen supply. Our study did not measure oxygen levels directly, but found evidence of reactive hyperemia. In another type of penile device, a vacuum compression device for erectile dysfunction, there have been no reports of persistent penile injury where a marked reperfusion hyperemia could be anticipated.^21^ Further research is required to assess the cumulative effect on tissues of continued PCD use.

The inflammatory response to applied compression was assessed by the up-regulation of the primary cytokine, IL-1α, which is secreted by keratinocytes in response to skin inflammation. IL-1α was rapidly released at the surface of the stratum corneum and collected in the sebum. Although a degree of inter- and intra-participant variability was evident, the trends in the IL-1α secretion profiles were similar (Figure 6). Increased IL-1α up-regulation was evident from baseline to 10 mins, ie, immediately after PCD application, plateauing by 50 mins coincident with two of the PCDs, the Uriclak and, to a lesser extent, the Dribblestop, resulting in an increased pressure over wear time (Figure 3). It is noteworthy that penile skin is particularly sensitive to mechanical loading as reflected in high concentrations of IL-1α in sebum when compared to that released from facial or forearm skin.^22^ No significant differences were seen between baseline levels and the values of IL-1α after a 40 mins recovery period.

For practical reasons, the test protocol was limited to a wear period of 50 mins. It is accepted that in normal use, individuals are likely to leave the PCD in situ for longer periods. Extended wear times might result in the development of superficial pressure ulcers (SPU) caused by elevated interface pressures, in conjunction with adverse microclimate conditions, typically, elevated skin surface temperature, humidity and air movement^23^ and it is, for example, well established that moisture at the device–skin interface increases friction forces.^24^

The study only included six participants. Considerable variability occurred between both individuals and the different PCDs. This is likely to be a reflection of several factors including the different PCD designs, the variability of male anatomy, skin sensitivity and the viscoelastic behavior of soft tissues.^19^ Despite this small number of and high variability between and within participants, objective methods that demonstrated physiological response to a relatively short period of PCD wear were validated. Whether a pressure ulcer would form with extended wear times remains unknown,^25^ due to the lack of published data for penile skin and the short wear period observed in this study.

## Conclusion

PCDs have proved as useful medical devices for some men who suffer from urinary incontinence following prostatectomy. Little safety research exists to assure men and healthcare professionals that these devices are safe to use. In this study, tissue and blood flow were compromised and pro-inflammatory cytokines raised during the wear time of 50 mins, when a PCD was applied at a pressure adequate to maintain continence during brief activities. However, none of the PCDs caused sustained irritation or impaired blood flow and yielded good recovery 40 mins after their removal. An important question remains as to how long individuals can safely wear a PCD and, after removal, how soon it can be re-applied.

This research indicates that application of a clamp for one hour with an equal clamp free time before reapplication is likely to be safe. Longer periods are often recommended by manufacturers but have yet to be tested.
